# Exocarp Properties and Transcriptomic Analysis of Cucumber (*Cucumis sativus*) Fruit Expressing Age-Related Resistance to *Phytophthora capsici*

**DOI:** 10.1371/journal.pone.0142133

**Published:** 2015-11-03

**Authors:** Kaori Ando, Kevin M. Carr, Marivi Colle, Ben N. Mansfeld, Rebecca Grumet

**Affiliations:** 1 Program in Plant Breeding, Genetics and Biotechnology, Michigan State University, East Lansing, MI, 48824, United States of America; 2 Research Technology Support Facility, Michigan State University, East Lansing, MI, United States of America; University of Tsukuba, JAPAN

## Abstract

Very young cucumber (*Cucumis sativus*) fruit are highly susceptible to infection by the oomycete pathogen, *Phytophthora capsici*. As the fruit complete exponential growth, at approximately 10–12 days post pollination (dpp), they transition to resistance. The development of age-related resistance (ARR) is increasingly recognized as an important defense against pathogens, however, underlying mechanisms are largely unknown. Peel sections from cucumber fruit harvested at 8 dpp (susceptible) and 16 dpp (resistant) showed equivalent responses to inoculation as did whole fruit, indicating that the fruit surface plays an important role in defense against *P*. *capsici*. Exocarp from 16 dpp fruit had thicker cuticles, and methanolic extracts of peel tissue inhibited growth of *P*. *capsici* *in vitro*, suggesting physical or chemical components to the ARR. Transcripts specifically expressed in the peel vs. pericarp showed functional differentiation. Transcripts predominantly expressed in the peel were consistent with fruit surface associated functions including photosynthesis, cuticle production, response to the environment, and defense. Peel-specific transcripts that exhibited increased expression in 16 dpp fruit relative to 8 dpp fruit, were highly enriched (P<0.0001) for response to stress, signal transduction, and extracellular and transport functions. Specific transcripts included genes associated with potential physical barriers (i.e., cuticle), chemical defenses (flavonoid biosynthesis), oxidative stress, penetration defense, and molecular pattern (MAMP)-triggered or effector-triggered (R-gene mediated) pathways. The developmentally regulated changes in gene expression between peels from susceptible- and resistant- age fruits suggest programming for increased defense as the organ reaches full size.

## Introduction

Fruit development is typified by a progression from fruit set, to exponential fruit growth, maturation, and ripening. Morphological and transcriptomic analyses of early cucumber fruit growth indicate that the period spanning anthesis through the end of exponential expansion is marked by two developmental transitions, one at the onset of exponential growth, the second at the end of exponential growth [1]. The first several days post-pollination (0–4 dpp), prior to exponential growth, are characterized by extensive cell division [1–3]. Transcripts nearly exclusively expressed during this time period include homologs of genes associated with cell cycle and DNA replication [1,4]. The first transition, as is typical of fruit development in general [5], is from cell division to cell expansion. Rapid fruit elongation in cucumber occurs from approximately 4–12 dpp [2,3,6,7]. Genes with peak expression at mid-exponential growth (8 dpp), included genes encoding cytoskeleton, cell wall, cuticle, and phloem-specific proteins [1,6]. A second shift in gene expression occurred at the end of exponential growth, 12–16 dpp, but well before the transition to maturity and ripening that occurs at about 25–30 dpp.

The transition accompanying the late/post-exponential growth stage, which has received little attention in the literature, was marked by strong enrichment for abiotic and biotic-stress related genes and induction of stress-related and development-related transcription factor gene homologs [1]. In certain cultivars, this time period is also associated with transition from susceptibility to resistance to an important cucumber disease, fruit rot caused by *Phytophthora capsici*. The soil borne oomycete pathogen, *P*. *capsici*, causes severe yield and economic losses for a variety of important vegetable crops, including cucumber (*Cucumis sativus*) [8,9]. The primary infectious agents responsible for spread of disease during the growing season are motile zoospores, which are released from asexual sporangia upon contact with water. For cucumber plants, it is primarily the fruit, rather than the leaves and vines that become infected [10]. Our prior studies showed that that very young cucumber fruit are highly susceptible, but at 10–12 dpp transition to resistance, becoming fully resistant by 16 dpp [10,11]. The transition to resistance was observed in bee-pollinated field-grown fruit, hand-pollinated greenhouse-grown fruit, and fruit that set parthenocarpically in the greenhouse, indicating that the resistance did not depend on the presence or development of seeds within the fruit [10,12]. An age-related reduction in susceptibility to *P*. *capsici* also was observed in fruit of several other cucurbit crops, including pumpkin, butternut squash, and acorn squash, although the effect was most pronounced for cucumber [11,13]. Age-related, or ontogenic, resistance also has been observed in strawberry fruit and grape berries in response to powdery mildew (*Podosphaera aphanis and Erysiphe nectator*, *respectively*) [14,15] and for apple fruit resistance to scab (*Venturia inaequalis*) [16].

In addition to the above examples in fruit systems, age-related resistance (ARR) has been observed in a number of other host-pathogen systems in a variety of tissue types, thus ARR is becoming increasingly recognized as an important component of plant defenses against fungal, oomycete, bacterial and viral pathogens [17–20]. However, the specific mechanisms responsible for resistance are just beginning to be explored and appear to vary among systems. By definition, ARR results from developmentally regulated changes that occur prior to contact with the pathogen, possibly by physical or chemical barriers, or by priming the organ for more rapid detection or response to infection. In several cases, ARR coincides with a developmental transition such as transition to flowering or critical leaf number as occurs for resistance of tobacco (*Nicotiana tabacum*) to *P*. *parasitica*, Arabidopsis to *Pseudomonas syringa* pv. *tomato*, and maize to *Puccinia sorghi* [21–24].

As the fruit surface is the first point of contact between the host and pathogen, in this study we sought to examine the role of the cucumber fruit surface in ARR to *P*. *capsici*. Tests with cucumber peels at different ages indicated a critical role of the fruit exocarp in expression of ARR, including possible biochemical defenses. Transcriptomic analysis of fruit peels identified developmental changes in gene expression in the surface tissue of resistant-age fruit. Peels from resistant-age fruit exhibited specific increase in transcripts associated with potential physical barriers, chemical defenses, and pathogen recognition responses.

## Materials and Methods

### Fruit peel experiments

Cucumber plants (pickling type, cv. Vlaspik; Seminis Vegetable Seed Inc, Oxnard, CA) were grown in greenhouse facilities at Michigan State University. No permits were required. Plants were grown in 3.78 L plastic pots filled with BACCTO (Michigan Peat Co., Houston, TX) or Suremix Perlite (Michigan Grower Product, Inc., Galesburg, MI) soil medium and fertilized once per week. Temperature was maintained at 21–25°C; supplemental lights were used to provide an 18 h light period. Pest control was performed according to standard management practices. Sets of 20–25 flowers were hand-pollinated on two dates to provide 15–20 fruit of each age to be harvested on the same day. To avoid competition between fruits, only one fruit was set per plant. The experiment was repeated three times.

The harvested fruit were washed, surface sterilized by brief immersion in a 5% sodium hypochlorite solution, rinsed with water several times, and allowed to air dry. Zoospore suspensions were prepared from 7-day old cultures of *P*. *capsici* isolate OP97 [10] grown on diluted V8 media and flooded with 6–10 ml sterile distilled water to release zoospores as described by Gevens et al. [10].

Preparation of zoospores of *P*. *capsici* isolate OP97 was performed according to Gevens et al. [10]. Concentration of the zoospore suspensions was determined by hemacytometer and diluted to 1 x 10^5^ per ml. Exocarp sections (3 cm x 3 cm x 1–2 mm) from the middle part of the fruit were excised from both 8 and 15 dpp fruit with petit knife or razor blade without introducing nicks. A sterile plastic tube (0.6 cm height, 0.8 cm diameter) was placed on the exocarp section and anchored to underlying intact 8 or 16 dpp fruit using a strip of 1 cm wide parafilm. A twenty-two gauge sterile needle was used to deliver 30 μl zoospore suspension into the tube by penetrating the parafilm; the needles did not come in contact with the fruit tissue. Inoculated fruit were incubated under constant light at 23–25°C in plastic wrap covered trays lined with moist paper to maintain high humidity. Intact 8 and 15 dpp fruits were similarly inoculated and included in each tray as control. Each tray contained each treatment combination: 8 day peel/8 day fruit; 8 day peel/15 day fruit; 15 day peel/8 day fruit; 15 day peel/15 day fruit; intact 8 day fruit; intact 15 day fruit. The peel sections and underlying fruit were monitored daily for 10 dpi and scored for stage of disease progression (1 –no symptoms, 2 –water soaked, and 3 –sporulation). The experiment was repeated three times. Data were analyzed as a randomized complete block design by ANOVA using the SAS program 9.1 (SAS Institute Inc., Cary, NC) with mixed procedures. Each value is the mean of at least 9 peel sections or fruit ±se. Bars marked with different letters indicate significant difference by LSD, P<0.05.

### Peel extract experiments

**Fig 1:**
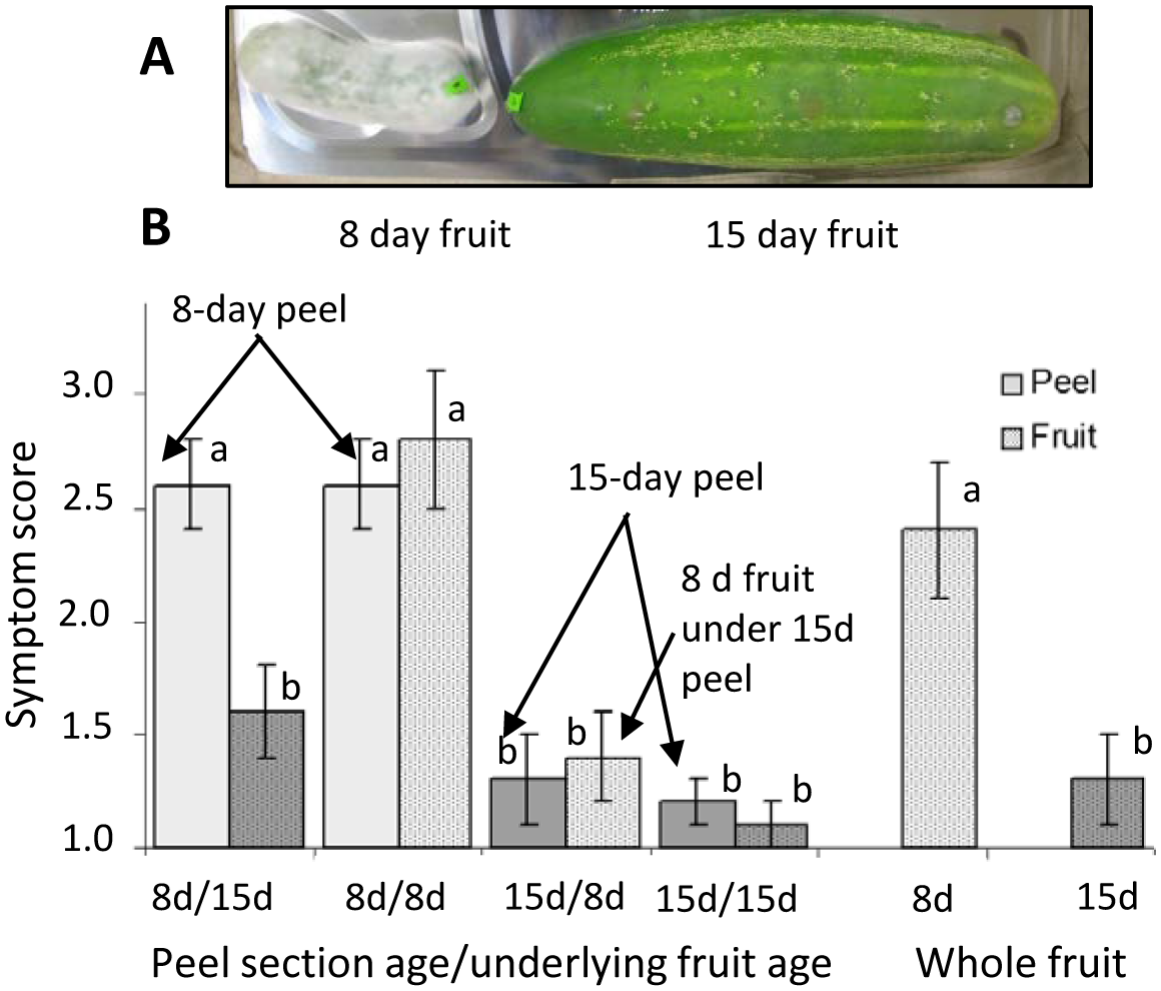
*Phytophthora capsici* disease development on cucumber fruit and peels. (A) Pathogen growth on whole, unwounded cucumber fruit. Fruit were harvested at 8 or 15 days post pollination (dpp) and inoculated with zoospore suspensions of *P*. *capsisi*. The photograph was taken at 5 days post-inoculation. The 8 dpp fruit is covered with mycelial growth. (B) Disease development on peel sections and underlying fruit, or directly inoculated whole fruit as described in methods. x/y indicates age of overlying peel section/age of underlying intact fruit. Symptom score: 1 = no symptoms or localized necrosis; 2 = water soaking; 3 = sporulation. Each value is the mean of at least 9 fruit ±se. Bars marked with different letters indicate significant different, ANOVA, LSD, P<0.05.

Fruit exocarp (1-2mm thick) was collected from the middle section of each fruit by razor blade. Frozen peel samples from fruits of the same developmental stage were pooled and used immediately for sequential extraction with water followed by methanol (Fig 1) based on the procedure by Jayaprakasam et al. [25]. Each extract was concentrated by rotary evaporation (BUCHI Rotavapor, BUCHI, Corp., Newcastle, DE) and freeze-dried using Genesis Pilot Freeze Dryer (SP Scientific Industries, Stoneridge, NY). The aqueous and methanolic extracts were redissolved in water and 10% methanol, respectively, to a final concentration of 25 μg ul^-1^. A 96-well clear (Thermo Fischer Scientific Inc., Waltham MA) or black microtiter plate (Griener Bio-One, Orlando, FL) was prepared with 200 μl clarified V8 media (centrifuged at 10,000 rpm for 10 min) per well. Samples were treated with 10 μl crude extract solution or solvent controls, and inoculated with 20 μl of 1x10^5^ zoospores ml^-1^ suspension of either *P*. *capsici* isolate OP97 or NY0664-1 expressing red fluorescent protein (RFP) ([26]; kindly provided by C. Smart, Cornell University) as described above. The inoculated plates were incubated at 25°C with a 16h light/ 8h dark cycle for 72 hours.

**Fig 2:**
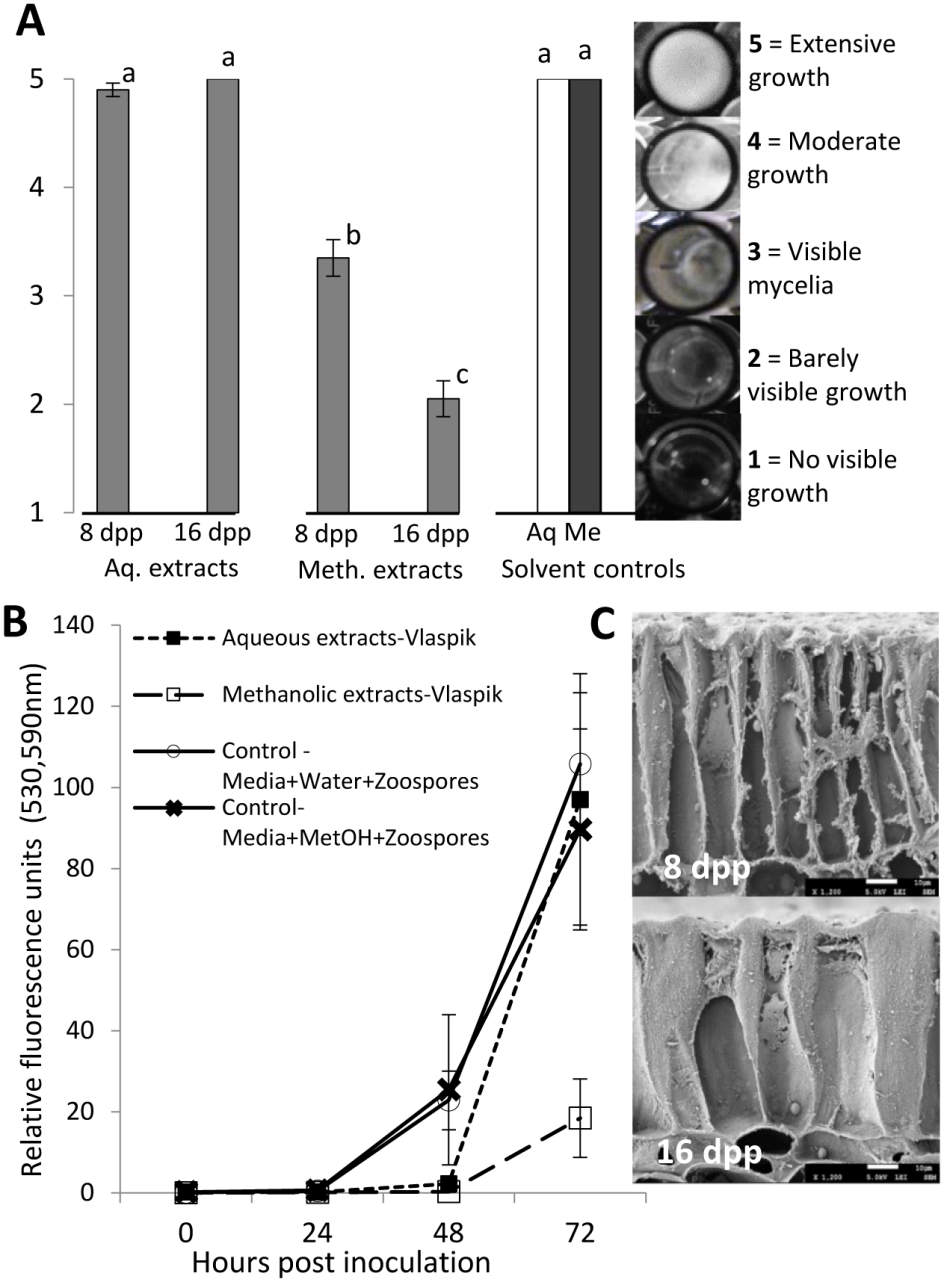
Chemical and physical properties of cucumber fruit peel at 8 and 16 days post pollination (dpp). (A, B) Effect of aqueous and methanolic extracts from cucumber fruit peel on growth of *Phytophthora capsici* *in vitro*. Media in individual wells were treated with 10 μl methanolic or aqueous extracts of cucumber fruit peel or solvent controls prepared as described in methods and inoculated with 20 μl of zoospores at 10^5^ zoospores ml^-1^. (A) Visual growth rating of isolate OP97 in response to extracts from 8 dpp and 16 dpp fruit peels, 3 days post inoculation. Rating was based on a 1–5 scale as illustrated in the sidebar. (B) Fluorescence emission from isolate NY0664-1RFP in response to aqueous and methanolic extracts. Each value is the mean of 4–5 replicate samples ± S.E. Bars marked with different letters are significantly different (LSD, P<0.05). Each experiment was performed twice with equivalent results. (C) Surface of cucumber fruit at 8 and 16 dpp. Magnification = 1200x; white bar = 10 microns.

Visual ranking was performed on a 1–5 scale at 3 dpi as illustrated in Fig 2A. Fluorescence values of the RFP-expressing cultures were measured at 530nm (excitation) and 590nm (emission) using SpectraMax M2e (Molecular Devices, Sunnyville, CA) at 0, 24, 48 and 72hrs post inoculation. Mean fluorescence measurements from the control (media with aqueous/methanolic extracts) were subtracted from the mean fluorescence values for the corresponding treatments. Samples within the plate were arranged in a randomized complete block design. Data were analyzed by ANOVA, followed by means separation by LSD, P<0.05. Each experiment was repeated two or three times with five replicate samples per treatment.

### Imaging of fruit exocarp by SEM

Sample preparation and imaging of cucumber fruit exocarp sections (2–3 mm) was performed by the Center for Advanced Microscopy of Michigan State University as briefly described here. Exocarp tissues were fixed in glutaraldehyde solution and dried in Balzers Model 010 critical point dryer (Balzers Union Ltd., balzers, Liechtenstein). After drying, the samples were mounted on aluminum stub using high vacuum carbon tabs (SPI supplies, West Chester, PA) and coated with osmium using a NEOC-AT osmium coater (Meiwafosis Co. Ltd., Osaka, Japan. Processed exocarp tissues were examined using a JEOL JSM-7500F scanning electron microscope (JEOL Ltd., Tokyo, Japan).

### Sample preparation for pyrosequencing

Cucumber plants were grown in the greenhouse as described above and in Ando et al. [1]. All flowers for each experiment were hand pollinated on a single date (1–2 flowers per plant). The experiment was repeated three times. Randomly assigned groups of twenty fruit were harvested at 8 and 16 dpp and ranked by size; the middle ten fruits were used for RNA extraction. Peel sections (1–2 mm thick) were removed by razor blade, immediately frozen in liquid nitrogen, and stored at– 70°C until RNA was isolated. Each biological replicate consisted of peel sections pooled from ten fruits; two biological replicates were prepared for each age. RNA extraction and oligo(dT)-primed cDNA sample preparation were based on the procedures of Schilmiller et al. [27] and Ando and Grumet [6]. Final concentration was assessed by the nanodrop ND-1000 Final concentration was assessed by the nanodrop ND-1000 (Thermo Scientific, Wilmington, DE) method and subsequent steps for 454 Titanium pyrosequencing analysis were performed by the Michigan State University Research Technology Support Facility (RTSF). Each sample was loaded on a 1/4 plate 454 Pico TiterPlate (454 Life Sciences, a Roche Corporation, CT). Pericarp samples consisting of exocarp, mesocarp, and placenta tissue but not seeds, from fruit grown at the same time in the greenhouse as those used for peel analysis, were sequenced previously [1].

### Contig assembly, EST mapping, and gene annotation

Contigs were assembled by the MSU RTSF Bioinformatics Group. Transcript assemblies were created from a collection of EST data sets from *Cucumis sativus*. An integrated pipeline was used to align individual reads to the *C*. *sativus* genome (ICuGI) using BLAT [28] and then to assemble clusters of overlapping alignments. The pipeline, Program to Assemble Spliced Alignments (PASA), is described in Hass et al. [29]. Prior to submitting the EST sequences to PASA they were cleaned using the TIGR SeqClean pipeline [http://compbio.dfci.harvard.edu/tgi/software/]. This was used to remove and vector or primer sequences, poly(A) tails and other low quality or low complexity sequences. Input to the PASA pipeline was comprised of 1.65 million reads generated from the nine libraries of ESTs from fruit at various developmental stages; all libraries were from fruit grown at the same time in the greenhouse. 99.2% of the ESTs were mapped to the genome. PASA assembled 53,677 putative transcripts clustered at ~32,000 loci on the genome. Read data for 8 day post pollination samples is available from the Sequence Read Archive (SRA), accessible through NCBI BioProject ID PRJNA79541. Read data for 0, 4, 12 and 16 dpp samples and the 8 and 16 dpp peel samples in SRA as well as assembled contig sequences deposited as Transcriptome Shotgun Assemblies (TSA) and expression profiling data in the Gene Expression Omnibus (GEO) are available through NCBI BioProject ID PRJNA169904 and DDBJ/EMBL/GenBank under the accession GDIL00000000.

Putative transcripts were annotated by BLAST comparison to both the Arabidopsis proteome (TAIR9) and the NCBI RefSeq Plant database; 37,800 putative transcripts scored a significant (e-value ≤ 10^−10^) hit to TAIR9 and 40,000 to RefSeq Plant. To estimate relative expression, the number of reads originating from each cDNA library were counted for each contig and reported relative to the total number of reads generated for that library as transcripts per hundred thousand (TPHT).

### Transcriptome analysis

The Classification SuperViewer Tool w/Bootstrap web database [30] was used for GO categorization, determination of normalized frequencies relative to Arabidopsis, and calculation of bootstrap standard deviations, and P-values. Princomp procedure SAS 9.1 (SAS Institute, Cary, NC) was used for principal component analysis. The first two principal components, which explain nearly 90% of the total variation were extracted from the covariance matrix. To identify transcripts either preferentially or minimally expressed in peel tissue, the proportion of reads obtained from the peel samples was calculated for each transcript for which there were ≥30 reads [i.e., reads from peel samples at 8 and 16 dpp/total reads (peel + pericarp) at 8 and 16 dpp]. Transcripts with increased expression in 16 dpp peel were identified by the ratio of reads from 16 dpp peel vs. 8 dpp peel. Putative cucumber homologs of the Arabidopsis SYP121/SNP33 regulon [31] were identified within the cucumber fruit transcriptome set and tested for co-expression with cucumber SYP121 and SNP33 by correlation analysis of transcript frequency in peel and pericarp across fruit age.

### qRT-PCR

Cucumber fruit used for qRT-PCR analysis were grown as described above for fruit peel experiments and pollinated on two dates, 8 days apart. Five fruits of each age (8 and 16 dpp) were harvested and quickly processed for RNA isolation. Peduncle and blossom ends were removed and peels separated from pericarp of the middle 5–10 cm of fruit tissue using a razor blade. Samples were quickly frozen in liquid nitrogen and stored at -80°C. RNA extraction and oligo(dT)-primed cDNA sample preparation were as described above. qRT-PCR primers were designed using NCBI Primer-BLAST [http://www.ncbi.nlm.nih.gov/tools/primer-blast] and tested for product specificity and reaction efficiency (S1 Table). qRT-PCR reactions were performed with the ABI Prism 7900HT Sequence Detection System (Life Technologies, Inc., Gaithersburg, MD). Samples were prepared using the rEVAlution Master Mix (Syzygy Biotech, Grand Rapids, MI) with ROX reference dye (Syzygy Biotech, Grand Rapids, MI) according to manufacturer’s instructions. Three technical replicates were prepared for each of the five peel and pericarp samples at each age. *C*. *sativus Ubiquitin 3 (CsUBQ3)* was used as an endogenous control. For standard curve dilutions a pool of 2 μl from each of the cDNA samples was collected and diluted to 20, 4, 0.8 and 0.16 ng/μl. PCR conditions were 50°C for two minutes, 95°C for 10 minutes enzyme activation, then 40 cycles of 95°C for 15 seconds and 60°C for 1 minute. Samples were quantified using the relative standard curve method for each gene. Relative quantification values (RQ) were normalized to the concentration of *CsUBQ3* in each sample.

## Results

### Cucumber fruit surface is an important determinant of ARR to *P*. *capsici*

With the exception of pathogens that enter through wounds or are delivered by a vector, the outer surface of a plant organ is typically the first point of contact with the host. We therefore sought to determine whether the ARR of cucumber fruit to infection by *P*. *capsici* was influenced by the fruit surface. Preliminary tests showed that when 16 dpp fruits were peeled prior to inoculation, 100% formed sporulating lesions, whereas none of the intact control fruit at 16 dpp become infected. While these results suggest that the fruit surface plays an important role in the resistance of older fruits to infection by *P*. *capsici*, it is also possible that the observed infection was facilitated by wounding as has been observed in other systems.

To eliminate possible confounding effects of injury, exocarp sections from fruits at 8 dpp or 15 dpp were placed on top of a second, intact fruit, and then inoculated with *P*. *capsici*. The peel sections responded equivalently to intact fruit (Fig 1). Like whole 8 dpp fruit, the 8 dpp peel pieces exhibited either water-soaking or sporulation, regardless of the age of the fruit underneath. Similarly, peel sections from 15 dpp fruit responded like intact 15 dpp fruit, regardless of fruit age underneath. In addition, even when subjected to the additional disease pressure of contact with an infected 8 dpp fruit peel, the underlying 15 dpp fruit did not typically become infected (8d/15d treatment). Finally, when 15 dpp fruit surface pieces were inoculated, the underlying 8 dpp fruit also maintained a very low disease score (15d/8d treatment), indicating that the 15 dpp fruit surface sections protected the underlying 8 dpp fruit. These results suggest that the surface of 15–16 dpp fruit possesses properties that inhibit *P*. *capsici* infection.

Surface factors influencing resistance may include biochemical or structural components. Cucumber leaves have been found to produce methanol-soluble phenolic and flavonoid compounds with antimicrobial properties [32–34]. Therefore we sought to test whether cucumber peels might also produce compounds with antimicrobial activity. Testing of aqueous and methanol extracts from peels of fruit at different ages showed that methanolic extracts from cucumber fruit peels inhibited growth of two *P*. *capsici* isolates in vitro (Fig 2A and 2B). Methanolic extracts from 16 dpp peels provided greater inhibition than from 8 dpp peels. Structural changes in the 16 dpp peels included thicker epidermal cell walls, increased cuticle thickness, and increased intercalation of cutin and waxes between adjacent cells in the epidermal layer (Fig 2C).

### Gene expression in cucumber fruit peel

To examine changes in gene expression specifically occurring in cucumber peels between fruit at susceptible and resistant ages, cDNA libraries were prepared from peel samples from 8 and 16 dpp fruit. Each biological replicate consisted of peel sections pooled from ten fruits; two biological replicates were prepared for each age. Pyrosequencing analysis yielded 814,250 ESTs from peel samples, which were combined with ESTs obtained from pericarp samples from 0, 4, 8, 12 and 16 dpp fruit that had been grown in the greenhouse at the same time [1], providing a data set of 1.65 million reads. Of those, 99.2% were mapped to the cucumber draft genome [35] at 38,318 loci.

The number of ESTs per assembled transcript ranged from 2–16,817 with a mean of 76 reads/transcript and a median of 9 reads/transcript. As was observed by Ando et al. [1], average assembled transcript length increased with the number of ESTs/transcript until approximately 30 ESTs/transcript (S2 Fig). At ≥30 reads/transcript the average length leveled off at approximately 1.6 kb/transcript. Significant BLAST hits to the NSCI RefSeq Plant gene database (E-value ≤ 10^−10^) were obtained for 74.5% of the assembled transcripts. Increasing number of ESTs/transcript was also associated with increased identification of putative homologs, again leveling off at approximately 30 ESTs/transcript (S2 Fig). Putative homologs were identified for 97% of the transcripts represented by ≥30 ESTs. Based on the observed relationship between ESTs/transcript, transcript length and identification of putative homologs, all subsequent bioinformatic analysis were performed with the set of transcripts represented by ≥30 ESTs (16,176 transcripts).

**Fig 3:**
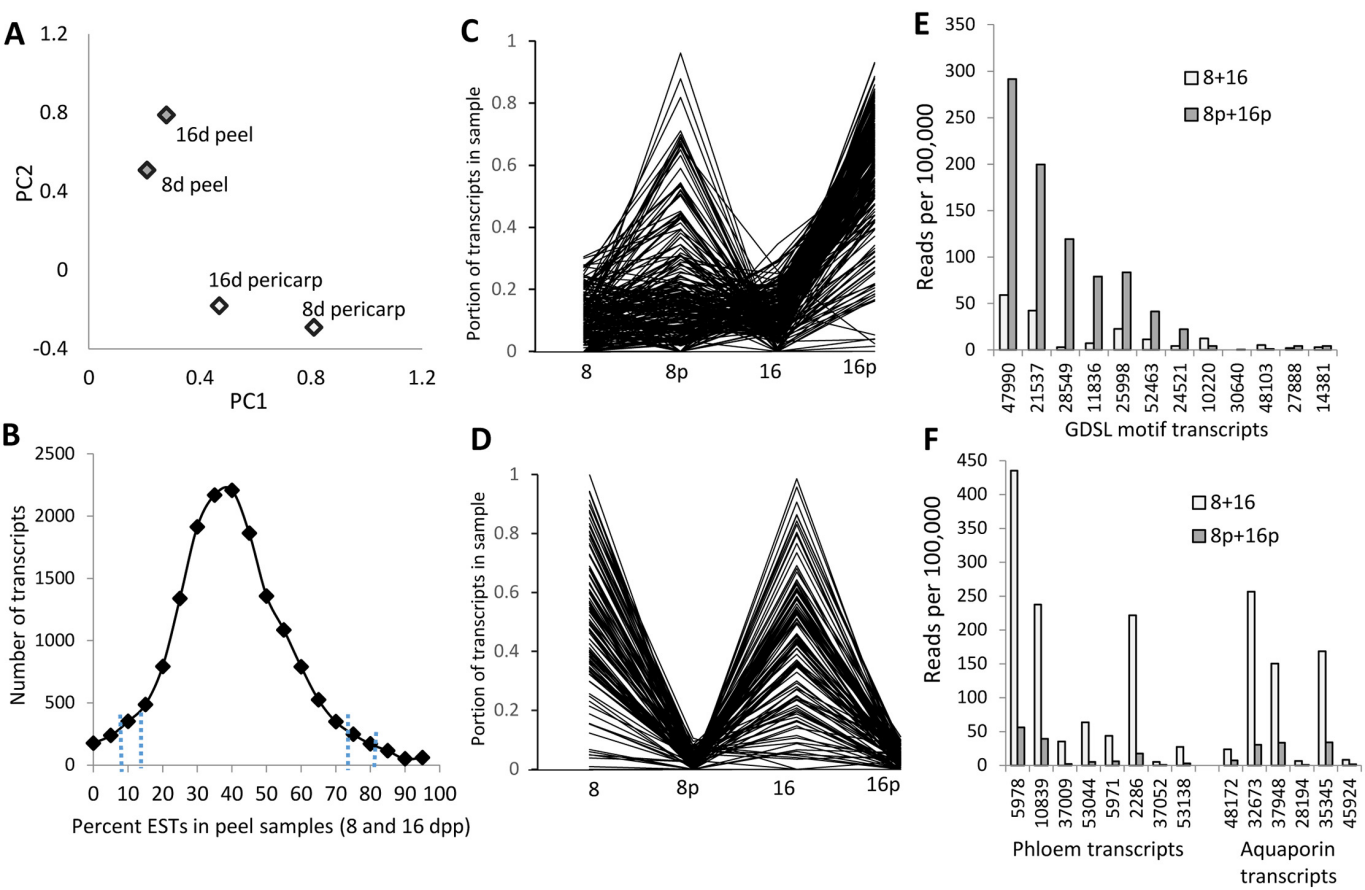
Gene expression in cucumber fruit peel and pericarp samples. (A) Principal component analysis of transcript expression levels for 8 and 16 days post pollination (dpp) peel and pericarp samples for all transcripts with ≥30 reads. The first two principal components account for 88% of the total variation. (B) Distribution of the portion of gene expression observed in the peel of 8 and 16 dpp fruit for all transcripts with ≥30 reads. The area to the right or left of the dotted vertical lines demarks those transcripts most strongly or most weakly expressed in the peel (top and bottom 2.5% and 5% respectively). (C, D) Expression patterns relative to fruit age and tissue type of genes preferentially expressed in (C) or excluded from (D) the peel at 8 and/or 16 dpp (8 = 8 dpp pericarp; 8p = 8 dpp peel; 16 = 16 dpp pericarp; 16p = 16 dpp peel). (E, F) Examples of transcripts differentially expressed between the peel (8p, 16p) and pericarp (8, 16) samples. (E) GDSL-motif transcripts. (F) Phloem and aquaporin transcripts. Gene labels refer to cucumber transcript assembly numbers (Sup. File 1).

Principal component analysis indicated that pericarp or peel samples at the different ages were more closely associated with each other than were pericarp and peel at the same age, indicating more commonality based on tissue type than fruit age (Fig 3A). To identify transcripts either preferentially or minimally expressed in peel tissue, the proportion of reads obtained from the peel samples relative to pericarp was calculated for each transcript as: reads from peel samples 8 and 16 dpp / total reads (peel + pericarp) at 8 and 16 dpp (Fig 3B). The proportion of reads obtained from the peel samples was approximately normally distributed over the population of transcripts. Genes present in the tails of the distribution, i.e., the top or bottom 5% (Fig 3C and 3D) showed functional differentiation. The 5% of transcripts with highest proportion of expression in the peel relative to the pericarp (a set of 813 transcripts for which >72% of the reads for each transcript were obtained from one or both of the peel samples), were specifically significantly enriched for cellular component categories of extracellular, ER, cell wall and plastid-related genes (S2 Fig). For example, cuticle-related transcripts, such as GDSL motif lipase gene homologs, were primarily expressed in the peel while phloem and aquaporin related gene homologs had minimal expression in the peel (Fig 3E and 3F)

Putative transcription factor genes that were primarily expressed in the peel (>80% reads from peel samples) included many that have been annotated to be involved in biotic and abiotic stress responses, including: *R2R3 MYB* domain proteins 30 and 96; *WRKY 15* and *40*; ethylene response factors (*ERF6*); heat shock factors (*HSFA3*); and salt tolerance zinc finger (*STZ*) factors (Table 1). The peel-expressed *MYB* factor genes all belong to R2R3 subgroup 1, which in Arabidopsis, is involved in drought stress and disease resistance functions including the ABA signal cascade, regulation of stomatal movement, and hypersensitive cell death response [36]. In contrast, expression of many development-related transcription factors observed in the pericarp, such as homologs of *AGAMOUS*, *Auxin response factor 4*, *FLOWERING LOCUS T*, *SEEDSTICK*, *SEPALLATA*, and *SHATTERPROOF 1* and *2*, were largely excluded from the peel samples.

**Table 1:** Putative transcription factors enriched or reduced in cucumber peel at 8 and 16 days post-pollination.

Transcript Number	Cucumber CDS	ESTs/100,000		% reads in peel	Hit ID Arabidopsis	Hit Description	E value
		Pericarp	Peel				
14161	3M710870.1	0.00	5.99	100.0	At1g80840	WRKY40 transcription factor	1.0E-77
9034	3M809420.1	1.07	7.95	88.2	At2g42200	SPL9 (SQUAMOSA Promoter binding protein-like 9)	1.0E-25
4603	5M603910.1	1.03	6.81	86.8	At1g10470	ARR4 (RESPONSE REGULATOR 4)	1.0E-62
29023	2M301540.1	1.07	6.77	86.4	At5g01880	Zn finger (C3HC4-type RING finger)	4.0E-33
9225	3M816030.1	8.96	52.03	85.1	At1g08810	MYB60 (myb domain protein 60)	4.0E-76
29496	2M354820.1	2.09	10.54	83.5	At1g27730	STZ (salt tolerance zinc finger)	2.0E-53
22834	6M094760.1	2.65	11.47	83.5	At5g50570	Squamosa promoter binding protein, putative	5.0E-43
15026	3M750350.1	1.03	5.17	83.3	At5g03720	At-HSFA3 (heat shock transcription factor A3)	2.0E-74
5285	2M428380.1	1.55	7.24	82.4	At2g23320	WRKY15; calmodulin binding	1.0E-69
42396	3M018320.1	4.17	19.11	82.1	At4g17490	AtERF6 (ethylene response element binding factor 6)	1.0E-52
43150	3M826690.1	2.65	11.40	81.1	At3g28910	MYB30 (myb domain protein 30)	9.0E-81
13524	1M033200.1	4.14	17.34	80.7	At5g62470	MYB96 (myb domain protein 96)	2.0E-89
7109	7M429520.1	2.59	10.41	80.1	At5g06710	HAT14 (Homeobox from Arabidopsis thaliana)	9.0E-79
14554	3M733980.1	7.36	0.00	0.0	At2g37740	ZFP10 (Zinc finger protein 10)	6.0E-19
41443	7M378520.1	29.80	0.00	0.0	At3g04730	IAA16; early auxin induced, transcription factor	1.0E-72
25851	Chrom5NA^a^	7.86	0.00	0.0	At4g09960	STK (SEEDSTICK)	4.0E-54
2743	6M520410.3	10.46	0.00	0.0	At4g18960	AG (AGAMOUS)	5.0E-45
42893	1M467100.1	84.30	0.55	0.7	At2g42830	SHP2 (SHATTERPROOF 2)	4.0E-74
38366	5M146260.1	9.46	0.25	2.6	At5g47610	Zn finger (C3HC4-type RING finger)	8.0E-35
50868	3M016400.1	7.78	0.21	2.7	At4g18020	APRR2 (pseudo response regulator 2)	1.0E-101
22831	6M095270.1	5.80	0.21	3.6	At1g24260	SEP3 (SEPALATTA3)	2.0E-65
48984	6M291920.1	17.25	0.78	4.3	At5g60450	ARF4 (Auxin response factor 4)	1.0E-129
22337	5M270900.1	16.64	0.75	4.3	At2g02070	AtIDD5 (Arabidopsis thaliana Intermediate domain 5)	1.0E-150
18472	1M651710.1	8.44	0.50	5.6	At1g65480	FT (FLOWERING LOCUS T)	2.0E-73
2433	6M526230.1	10.51	0.71	6.4	At2g34830	WRKY35 (WRKY DNA binding protein 35)	2.0E-71
25082	4M645240.1	10.07	0.96	8.7	At1g10120	DNA binding transcription factor	2.0E-63
24179	2M000630.1	28.67	2.78	8.8	At2g19810	Zn finger (CCCH-type family protein)	1.0E-101
51052	5M198240.1	18.85	1.99	9.6	At5g05790	MYB family transcription factor	3.0E-71
48460	3M073900.1	37.82	4.09	9.8	At1g50640	ERF3 (ethylene response element binding factor 3)	8.0E-61

^a^ Not annotated—located in cucumber genome, but not currently annotated in Chinese Long v.2 draft

Collectively, these observations reflect functional differentiation between the peel and pericarp. Transcripts predominantly expressed in the peel were consistent with fruit surface associated functions including photosynthesis, cuticle production, response to the environment, and defense.

### Transcripts expressed specifically in peel from 16 dpp fruit

We next sought to identify transcripts that were primarily expressed in the peel, and also showed increased expression in 16 dpp fruit relative to 8 dpp fruit, i.e., specifically expressed at 16 dpp rather than 8 dpp. The 105 transcripts that met both criteria (top 5% for expression in peel, and top 5% for increase in 16 dpp peel vs. 8 dpp peel) exhibited 8–800 fold enrichment in 16 dpp peel relative to 8 dpp peel (S2 Table). Of those transcripts, 9 did not have putative homologs in the NSCI RefSeq Plant gene database, and an additional 12 with homologs did not have functional annotation. Those with annotation showed strong enrichment for GO categories of response to stress, extracellular, response to abiotic or biotic stimulus, signal transduction, and transport functions (Table 2). The greatest reductions in expression in 16 dpp peel relative to 8 dpp peel were observed for the categories of chloroplast, and plastids. This observation is developmentally consistent with the reduced chlorophyll content observed at these ages [1].

**Table 2:** Functional enrichment of genes preferentially expressed in peels at 16 days post pollination (dpp). Transcripts were selected based on criteria of top 5% for expression in peel and top 5% for increase in 16 dpp peel vs. 8 dpp peel. The resulting 105 transcripts had >72% of reads in peel samples and >8-fold increase above 8 dpp peel samples.

Classification ^a^	Normalized frequency	Bootstrap Std Dev	P value
Other biological processes	3.09	0.357	1.13 E-11
Response to stress	2.70	0.334	3.89 E-10
Other enzyme activity	2.43	0.300	4.18 E-07
Extracellular	2.40	0.444	1.74 E-05
Response to abiotic of biotic stimulus	2.32	0.361	1.73 E-06
Signal transduction	2.29	0.456	6.81 E-04
Transport	1.99	0.328	2.28 E-04
Other membranes	1.64	0.295	5.23 E-03
Other metabolic processes	1.50	0.080	4.37 E-07
Other cytoplasmic components	1.40	0.19	9.28 E-03
Other cellular processes	1.36	0.083	6.77 E-05

^a^ Functional distribution, normalized frequency, and bootstrap standard deviation (SD) of contigs with putative Arabidopsis homologs was determined using the categories classification from the Classification SuperViewer from Bio-Array Resource for Arabidopsis Functional Genomics for Gene Ontology [http://compbio.dfci.harvard.edu/tgi/982 software96/].

**Fig 4:**
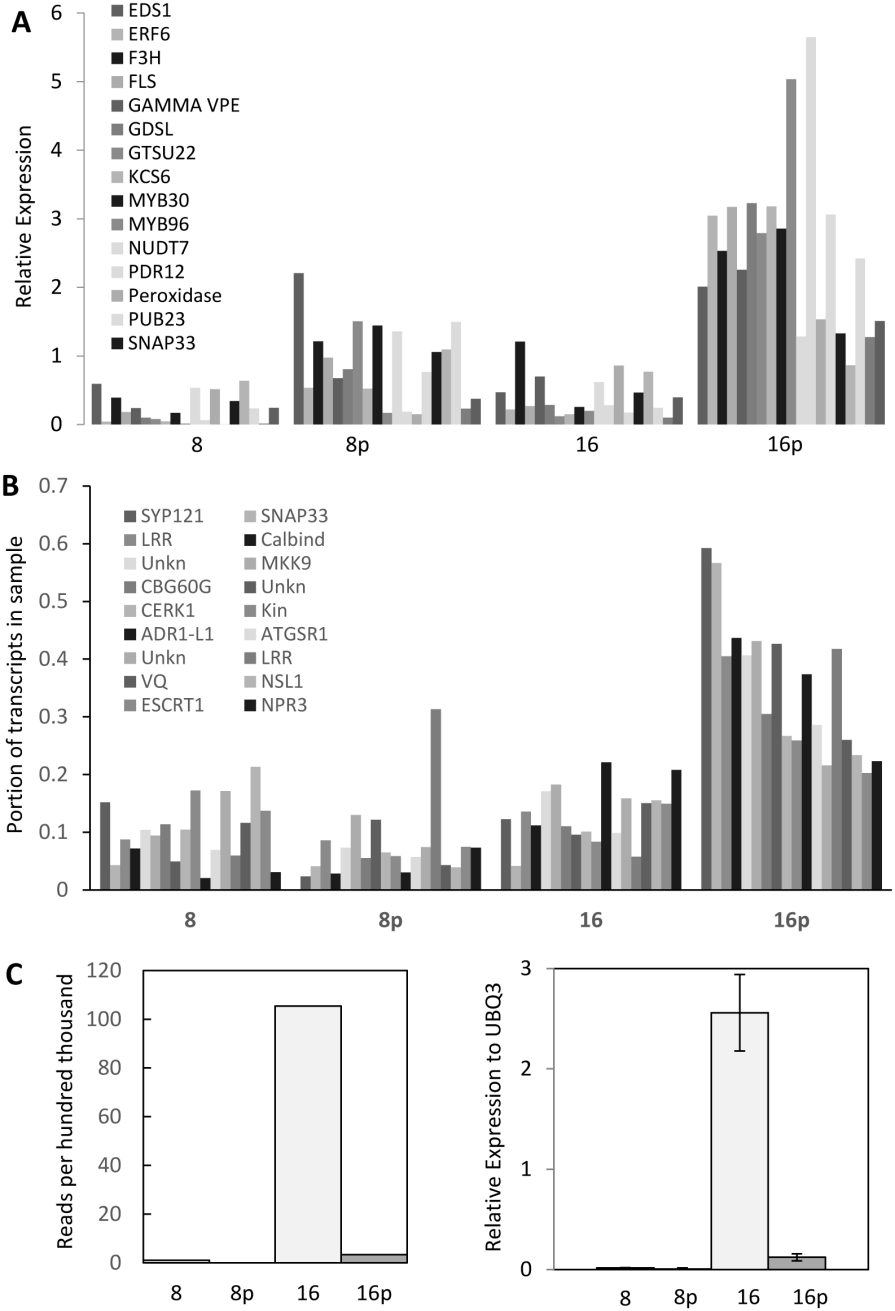
Expression analysis of putative defense related genes. (A) qRT-PCR verification of potential pathogen defense-related genes with elevated expression in 16 days post pollination (dpp) peels. (B) Relative expression of SYP121/SNAP33 co-expressed genes in 8 and 16 dpp pericarp and peel samples as assessed by 454 pyrosequencing. Genes shown are in the order listed in Table 4. (C) Expression of *CsFM01* as assessed by 454 pyrosequencing (left) and qRT-PCR analysis (right). 8 = 8 dpp pericarp; 8p = 8 dpp peel; 16 = 16 dpp pericarp; 16p = 16 dpp peel.

Greater than 40% of the 16 dpp peel-enriched genes were potentially associated with pathogen defense based on annotations from other systems (Table 3). A subset of 20 putative defense-related genes whose transcriptome patterns showed elevated expression in 16 dpp peel samples was selected for verification of expression by qRT-PCR analysis of pericarp and peel samples from 8 dpp and 16 dpp fruit (Fig 4A). The qRT-PCR results mirrored those of the 454 analysis, substantiating the 454 analysis, and also demonstrating reproducibility of gene expression patterns across experiments grown at different times in the greenhouse. Predominant expression was observed in 16 dpp peel tissue, although for a few genes (*EDS1*, *NUDT7*, *SYP121*), the difference between 8 dpp peel and 16 dpp peel was less pronounced in the qRT-PCR analysis than from the transcriptome data.

**Table 3:** Putative pathogen defense-associated transcripts preferentially expressed in cucumber fruit peel at 16 days post pollination.

Transcript Number	Cucumber CsaCDS	ESTs/10^5^ 16 dpp peel	% reads in peel	16dpp peel/8dpp peel	Hit ID Arabidopsis	Hit Description	E value
42396	3M018320.1	18.33	82.1	21.0	At4617490	ATERF6 (ethylene responsive element binding factor 6)	1E-52
53532	1M033160.1	17.01	87.0	27.3	At5g62480	ATGSTU9 (glutathione-S-transferase TAU 9)	1E-64
43980	4M064630.1	59.49	87.5	8.9	At1g78340	ATGSTU22 (glutathione-S-transferase TAU 22)	2E-42
43984	4M064650.1	33.46	73.9	16.7	At1g17180	ATGSTU25 (glutathione-S-transferase TAU 25)	1e-91
3557	6M507520.1	59.35	80.5	157.7	At3g12500	ATHCHIB (Arabidopsis thaliana basic chitinase)	1E-128
34558	7M031650.1	9.45	80.2	9.4	At4g12720	ATNUDT7 (nudix hydrolase homolog 7)	3E-86
24234	2M000460.1	2.85	72.8	29.5	At4g29720	ATPA05 (Polyamine oxidase 5)	1E-170
38780	4M622230.1	17.98	75.6	11.1	At3g14690	CYP72A15; electron carrier	1E-161
15574	1M006320.1	3.46	87.0	35.6	At3g48090	EDS1 (enhanced disease susceptibility 1)	1E-117
3105	6M516960.1	7/56	80.1	8.7	At4g21510	F-box family protein	2E-30
3642	6M507140.1	52.72	87.3	46.8	At1g75900	Family II extracellular lipase (EXL3)	1E-100
22665	6M108510.1	8.10	80.2	21.8	At3g51240	F3H (flavonone-3-hydroxylase)	1E-120
29623	2M354820.1	36.49	76.9	10.0	At4g32940	GAMMA-VPE (Gamma vacuolar processing enzyme)	0
21123	6M117710.1	6.71	86.9	18.1	At1g76490	HMG1 (hydroxy methylglutaryl CoA reductase 1)	0
4009	5M609650.1	71.39	83.6	38.5	At1g68530	KCS6 (3-ketoacyl-CoA synthase 6)	0
1636	3M144140.1	14.91	91.1	13.6	At5g43760	KCS20 (3-ketoacyl-CoA synthase 20); fatty acid elongase	0
13524	1M033200.1	16.51	80.7	17.9	At5g62470	MYB96 (MYB domain protein 96)	2E-89
10009	4M008780.1	8.67	1.00	13.4	At3g53260	PAL; phenylalanine ammonia-lyase	0
44827	2M433930.1	7.53	87.9	76.3	At1g59870	PEN3; PDR12 (PLEIOTROPIC DRUG RESISTANCE 12)	0
27490	4M285730.1	53.46	88.2	85.4	At5g06720	Peroxidase 2	7E-95
53444	4M285760.1	33.59	78.6	23.5	At5g06730	Peroxidase superfamily protein	3E-98
52067	6M213910.1	20.47	76.7	205.7	At5g05340	Peroxidase superfamily protein	1E-116
8758	3M782630.1	9.45	90.4	27.3	At2g35930	PUB23 (PLANT U-BOX 23) ubiquitin protein ligase	4E-123
13606	1M039020.1	14.12	87.8	12.6	At5g61210	SNAP33 (soluble N-ethylmaleimide-sensitive factor adaptor protein)	3E-91
50760	2M251460.3	3.85	79.4	11.3	At4g34640	SQS1 (squalene synthase 1)	0
25013	4M642460.1	41.05	77.5	21.9	At1g27730	STZ (salt tolerance zinc finger)	2E-58
29496	2M354820.1	9.98	83.5	15.4	At1g27730	STZ (salt tolerance zinc finger)	2E-53
8776	3M782680.1	6.95	92.9	70.5	At3g11820	SYP121 (SYNTAXIN OF PLANTS 121); PEN1 (penetration 1)	1E-69
34134	3M643770.1	8.74	90.1	10.1	At5g07990	TT7 (Transparent testa 7) flavonoid 3’-monoxygenase	1E-114
14161	3M710870.1	5.49	100.0	9.3	At1g80840	WRKY40 transcription factor	1E-77
47100	7M390100.1	7.92	77.2	8.6	At1g48910	YUC10; FAD binding/monooxygenase	7E-98
3738	6M505230.1	4.21	89.1	43.1	At2g21320	Zinc finger (B box type) family protein	2E-51

#### Age-related, peel-specific, gene expression potentially associated with biochemical and structural defenses

The 16 dpp peel samples were specifically enriched for expression of several groups of genes potentially associated with biochemical or structural defenses. These include putative homologs of genes encoding enzymes associated with flavonoid biosynthesis such as: phenylalanine amonia-lyase (*PAL*), flavanone-3-hydroxylase (*F3H*) and flavonoid 3'-monooxygenase (also flavonoid 3' hydroxylase; *TT7*) (Table 3). A putative homolog of flavonol synthase At5g08640 (1.0E-120) was also highly expressed in the peel (92% of transcript reads) with 4.7-fold higher expression in 16 dpp peel than 8 dpp peel.

Another group of genes with strong expression in the 16 dpp peel samples included members of the glutathione S-transferase (GST) gene family (Table 3). GSTs, which are highly expressed in plants and can comprise 1–2% of the soluble proteins, have been associated with a range of biotic stress response related functions, including increased resistance to several fungal or oomycete pathogens [37]. The GST family is classified into six groups (phi, tau, theta, zeta, lambda, and dehydroascorbate reductase) that exhibit tissue- and developmental- specific expression. The cucumber fruit transcriptome included twelve putative GST family members representing five of the six groups (Additional File 4). Three showed strong expression specifically in the 16 dpp peel samples (Table 3); all three were members of the tau group, which has been associated with resistance to biotic stresses [38, 39].

One of the genes with the strongest 16 dpp peel-specific expression was a putative member of the fungal and plant-specific, pleiotropic drug resistance (*PDR*) ATP-binding cassette (ABC) transporter sub-family. *CsPDR12* (*PEN3*) showed greater than 70-fold elevated expression in 16 dpp peel relative to 8 dpp peels, and essentially exclusive expression in 16 dpp peel as assessed by qRT-PCR analysis (Table 3, Fig 4A). ABC transporters have been implicated in transport of a wide variety of structurally unrelated molecules, including flavonoid related compounds [40–42].

The 16 dpp peel sample also showed peak expression of putative homologs of several cuticle associated genes [43, 44] including: two GDSL motif genes; two GDSL-like extracellular lipases; a long chain fatty acid- CoA ligase family protein gene; and two very long chain fatty acid synthesis related genes [KCS6 and 20 (3-ketoacyl-CoA synthase 6 and 20)] (Table 3; S2 Table; Fig 4A).

#### Age-related, peel-specific, gene expression of defense pathway associated genes

Several of the genes strongly represented in the 16 dpp peel samples are putative homologs of genes that have been specifically associated with microbial associated molecular pattern (MAMP)-triggered defense, suggesting possible priming for defense (Table 3). A central feature of MAMP defense is an oxidative burst resulting from rapid production of reactive oxygen species (ROS), including peroxidase-mediated production of hydrogen peroxide [45,46]. The oxidative burst can inhibit pathogen growth, signal induction of host defense responses, and promote hypersensitive response. The genes showing increased expression in the 16 dpp peel included putative homologs of three members of the peroxidase gene family with 20-200- fold up-regulation relative to 8 dpp peel. Peroxidases also have been implicated in additional roles that may contribute to plant defense, including strengthening of cell walls (e.g., protein cross linking, lignification), potentially inhibiting pathogen penetration [46].

Notably, two of the genes specifically highly expressed in the 16 dpp peel were putative homologs of genes that confer resistance to penetration of fungal and oomycete pathogens in Arabidopsis and barley, *SYP121 (SYNTAXIN OF PLANTS)*/*PEN1(PENETRATION1)* and *SNAP33* [47]. *SYN121/PEN1* function is associated with more rapid formation of penetration-resistant papillae structures containing callose, phenolic compounds, lignin, and reactive oxygen species [48]. As was observed for Arabidopsis and barley, the cucumber *SYP121* and *SNAP33* homologs were highly co-expressed (R = 0.976, P<0.001). In silico analysis of Arabidopsis genes identified a set of 107 genes co-expressed with *PEN1*, *SNAP33* and *MLO2* [31]. Putative homologs for 37 of these genes were observed in the cucumber fruit data set, of which 17 showed patterns of expression correlated with expression of the cucumber *SYP121/PEN1* and *SNAP33* homologs (Table 4; Fig 4B). These genes included putative homologs of the elicitor response genes, *CERK1* (chitin elicitor receptor kinase 1), and *MKK9* (MAP kinase kinase 9) [48]; SA-mediated and hypersenstitive response genes, *SARD1* (SAR deficient 1) and *CPB60G* [49]; the SA receptor, *NPR3* (*NPR1*-like gene 3; non-expressor of *PR* genes 1-like protein 3) [50], and a defense response regulator, *NSL1* (necrotic spotted lesions 1) [51].

**Table 4:** Co-expression analysis of putative cucumber homologs of MLO2/SYP121/SNAP33- co-expressed genes from Arabidopsis. Putative homologs of MLO2/SYP121/SNAP33- co-expressed genes from Arabidopsis (as identified by Humphrey et al.[31] were tested for co-expression with cucumber SYP121 and SNAP33 genes in cucumber pericarp (0, 4, 8, 12, and 16 dpp) and peel (8 and 16 dpp) samples.

TranscriptNumber	Cucumber Csa CDS	ESTs/10^5^ 16 dpp peel	Corr. With SYP121/SNAP33	P-value	Hit ID Arabidopsis	Hit Description	E value
8776	3M782680.1	12.5			At3g11820	SYP121 (Syntaxin of plants 121);PEN1 (penetration1)^b^	1.0E-124
13606	1M039020.1	14.1			At5g61210	SNAP33 (soluble N-ethylmaleimide-senstive factor adaptor protein 33)	3.0E-91
33242	1M601530.1	94.1	0.984	<0.001	At5g21090	Leucine-rich repeat protein, putative	1.0E-100
13843	1M042460.1	16.2	0.975	<0.001	At1g73805	Calmodulin binding; SARD1 (systemic acquired resistance deficient 1)	1.0E-94
14259	3M730710.1	27.1	0.963	<0.001	At5g01750	Unknown protein	9.0E-60
13997	1M042980.1	9.8	0.925	0.002	At1g73500	MKK9 (MAP kinase kinase 9)	1.0E-114
6185	1M569130.1	4.3	0.897	0.004	At5g26920	CBP60G (CAM-binding protein 60-like G); calmodulin binding	1.0E-51
8202	3M889130.1	9.2	0.876	0.005	At2g25737	Unknown protein	0.0
34393	7M041930.1	5.5	0.856	0.007	At3g21630	CERK1 (Chitin elicitor receptor kinase 1)	0.0
46112	3M651840.1	6.2	0.847	0.008	At3g14050	RSH2 (RelA SpoT homolog 2)	0.0
31565	2M070870.1	12.8	0.835	0.010	At2g24360	Serine/threonine/tyrosine kinase, putative	1.0E-117
24820	4M638480.1	9.6	0.833	0.010	At4g33300	ADR1-L1 (Activated disease resistance1-like 1); NB-LRR family protein	0.0
38247	3M304140.1	33.0	0.794	0.018	At5g37600	ATGSR1 (copper ion binding / glutamate-ammonia ligase)	0.0
38772	4M621210.1	14.8	0.793	0.018	At1g17080	Unknown protein	1.0e-44
47275	7M373520.1	3.7	0.734	0.031	At5g48380	Leucine-rich repeat family protein	4.0E-58
40525	1M074920.1	10.7	0.718	0.036	At1g28280	VQ motif containing protein	4.0E-66
40561	1M075570.1	4.7	0.689	0.044	At1g28380	NSL1 (necrotic spotted lesions 1)	2.0E-63
14951	3M746590.1	6.3	0.626	0.068	At2g36680	Located in ESCRT 1 complex	8.0E-71
43939	4M063470.1	3.9	0.614	0.073	At5g45110	NPR3 (NPR1-like protein 3), non-expressor of PR genes1-like protein3	1.0E-152

Putative homologs of genes associated with effector triggered-defense such as *EDS1* (*ENHANCED DISEASE SUSCEPTIBILITY 1*) and *NUDT7* (*NUDIX HYDROLASE HOMOLOG 7*) (Table 3) were also enriched in the 16 dpp peel samples. *EDS1* in Arabidopsis regulates R gene-mediated and systemic resistance, acting in combination with several other factors including *AtNUDT7* and the flavin-dependent monooxygenase, *FMO1*, to regulate cell death responses [52,53]. A homolog of *FMO1* (At1g19250, 1.0E-116) was minimally expressed in cucumber fruit samples at 0, 4 or 8 dpp, but then increased 50- and 100-fold at 12 and 16 dpp, respectively. The transcripts, however, were almost exclusively located in the pericarp (97%) samples, rather than peel (Fig 4C), suggesting that if FMO1 plays a role in resistance to *P*. *capsici*, it likely occurs at an infection step post-penetration.

The set of 16 dpp peel-enriched genes also included putative homologs of R2R3 subgroup 1 MYB transcription factor MYB 96 and a vacuolar processing cysteine protease enzyme, Gamma-VPE, which is a critical component of the hypersensitive programmed cell death response (Table 3) [36,54]. The peel-expressed putative homologs of abiotic and biotic stress MYB factor 96 was 18 fold up-regulated in the 16 dpp peel relative to 8 dpp peel (Table 3), while MYB30, which in Arabidopsis has been found to encode an activator of the hypersensitive cell death response via regulation of 402 biosynthesis of very long chain fatty acids [55], exhibited 4.1-fold increase in 16 dpp peel relative to 8 dpp peel (Table 1, S2 Table).

## Discussion

These studies showed that ARR of cucumber fruit to infection by *P*. *capsici* is associated with the fruit surface. Peel sections from 16 dpp fruit exhibited resistance, while peel sections from 8 dpp fruit were highly susceptible. We therefore sought to examine changes in the fruit peel that might contribute to increased resistance. Peel sections from 16 dpp had obvious differences in surface morphology and produced increased levels and/or types of methanol-soluble compounds capable of inhibiting growth of *P*. *capsici* in vitro. Transcriptome analysis reflected functional differentiation for gene expression between the peel and pericarp with increased expression of fruit surface associated functions such as photosynthesis, cuticle production, response to the environment, and defense in the peel tissue. Gene expression that was specifically associated with peel sections from resistant age fruit showed strong enrichment for transcripts annotated to be associated with response to stress or abiotic or biotic stimuli, signal transduction, and transport and extracellular functions. Greater than 40% of transcripts of the 16 dpp peel-enriched genes were potentially associated with pathogen defense.

Consistent with methanol-soluble compounds capable of inhibiting growth of *P*. *capsici*, was increased expression of homologs of several genes associated with flavonoid and phenylpropanoid biosynthesis, *PAL*, *F3H*, *TT7* and *FLS*. Previous studies have observed phenylpropanoid-derived phenolics, *C*-glycosyl flavonoids, and aglycones associated with resistance to powdery mildew (*Podosphaera xanthii*) in cucumber leaves [32–34] and inhibitory glycoside-linked phenolic compounds that increase with leaf age, have been found to localize to cucumber leaf cells beneath penetrating appressoria of *Colletotrichum orbiculare* [56]. Such defensive compounds may serve roles as phytoanticipins, accumulating prior to infection, or may exhibit increased resistance in response to infection. The developmental regulation of expression of phenylpropanoid-associated genes, and the presence of pathogen-inhibitory, methanol-soluble compounds in the 16 dpp peel, suggests synthesis of preformed chemical barriers. However, induced resistance in cucumber leaves to powdery mildew has been found to be dependent on elevated activity of flavonoid pathway enzymes [32], suggesting potential for induced response as well. Increased expression of PAL, a key upstream enzyme for flavonoids, as well as salicylic acid and lignin biosynthesis, has been observed in a wide range of systems, including cucumber and melon, in response to treatment with pathogens or chemical inducers (e.g., [57,58]).

The 16 dpp fruit peel sections also had specific elevation of expression of several *GST* genes and the pleiotropic drug resistance gene family member homolog, *CsPDR12*. Members of both *GST* and *PDR* gene families can facilitate export of flavonoid and terpenoid related compounds to the cell wall where they can accumulate in the cuticle [59,60]. The *PDR12* homolog from *Nicotiana plumbaginifolia* (*NpPDR1*), shows age-related and epidermal-specific transcription and exports an anti-fungal/oomycete terpenoid to the leaf surface [41,61]. Several members of the Arabidopsis *PDR* family show age-specific expression in developing seedlings [62].

Other types of defense related genes that were specifically, highly expressed in the peel of 16 dpp fruit were homologs of genes that have been associated with resistance to fungal and oomycete penetration by more rapid formation of papillae, as well as numerous putative elicitor-, effector-, and SA- response genes which may play roles in MAMP or R-gene mediated resistance. While at this time, we cannot determine whether these genes are specifically associated with ARR to *P*. *capsici*, the transcriptomic analysis indicating increased expression of a large number of putative-defense related genes, raises the possibility that ARR results from systematic, developmental reprogramming for defense.

In summary, analysis of cucumber fruit indicated importance of the fruit surface for ARR to *P*. *capsici* and a potential role for methanol-soluble inhibitory compounds. Transcriptomic studies of the fruit peel suggest developmentally-regulated expression of defense genes potentially associated with structural, biochemical (flavonoid pathway and transporters), MAMP response, and effector-triggered or R-gene mediated resistances.

## Supporting Information

S1 Fig Relationship between number of ESTs per contig and mean contig length (A) or % of contigs with homologs in Arabidopsis (B). (PPTX)

S2 Fig GO enrichment analysis for transcripts preferentially expressed or preferentially excluded from the peel. (PPTX)

S1 Table Primers used for qRT-PCR analysis. (DOCX)

S2 Table List of transcripts preferentially expressed in cucumber fruit peel at 16 days post pollination (dpp). (XLSX)
