# Supplementary material for: Exocarp Properties and Transcriptomic Analysis of Cucumber (Cucumis sativus) Fruit Expressing Age-Related Resistance to Phytophthora capsici
Source: PLoS One. 2015 Nov 3;10(11):e0142133. doi: 10.1371/journal.pone.0142133 (PMC4631441; doi:10.1371/journal.pone.0142133)
Supplement: S1 Fig — (PPTX) [file pone.0142133.s001.pptx]

## Slide 1
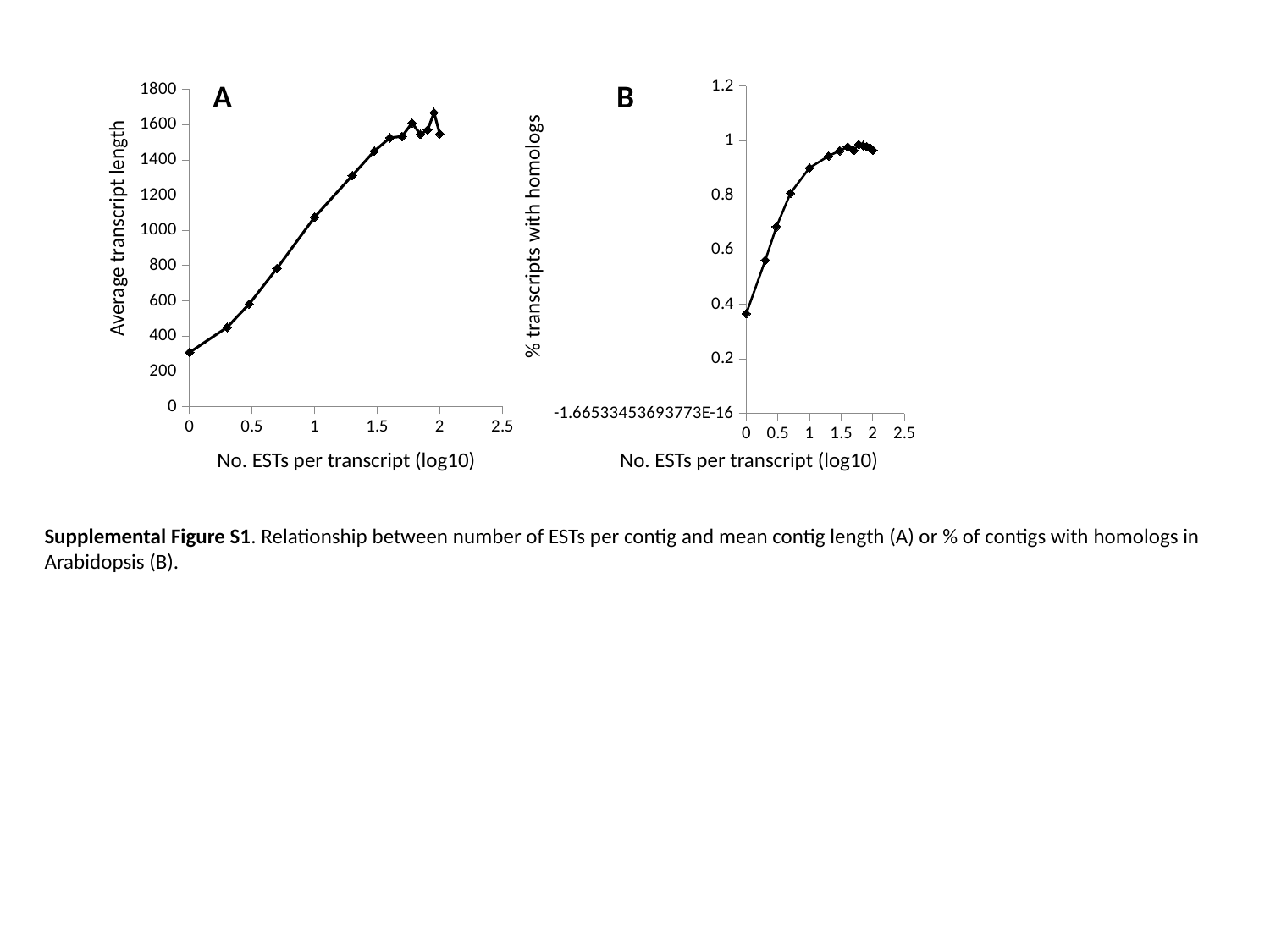

B
### Chart
| Category | |
|---|---|
### Chart
| Category | |
|---|---|Average transcript length
% transcripts with homologs
No. ESTs per transcript (log10)
No. ESTs per transcript (log10)
A
Supplemental Figure S1. Relationship between number of ESTs per contig and mean contig length (A) or % of contigs with homologs in Arabidopsis (B).
