# Supplementary material for: Exocarp Properties and Transcriptomic Analysis of Cucumber (Cucumis sativus) Fruit Expressing Age-Related Resistance to Phytophthora capsici
Source: PLoS One. 2015 Nov 3;10(11):e0142133. doi: 10.1371/journal.pone.0142133 (PMC4631441; doi:10.1371/journal.pone.0142133)
Supplement: S2 Fig — (PPTX) [file pone.0142133.s002.pptx]

## Slide 1
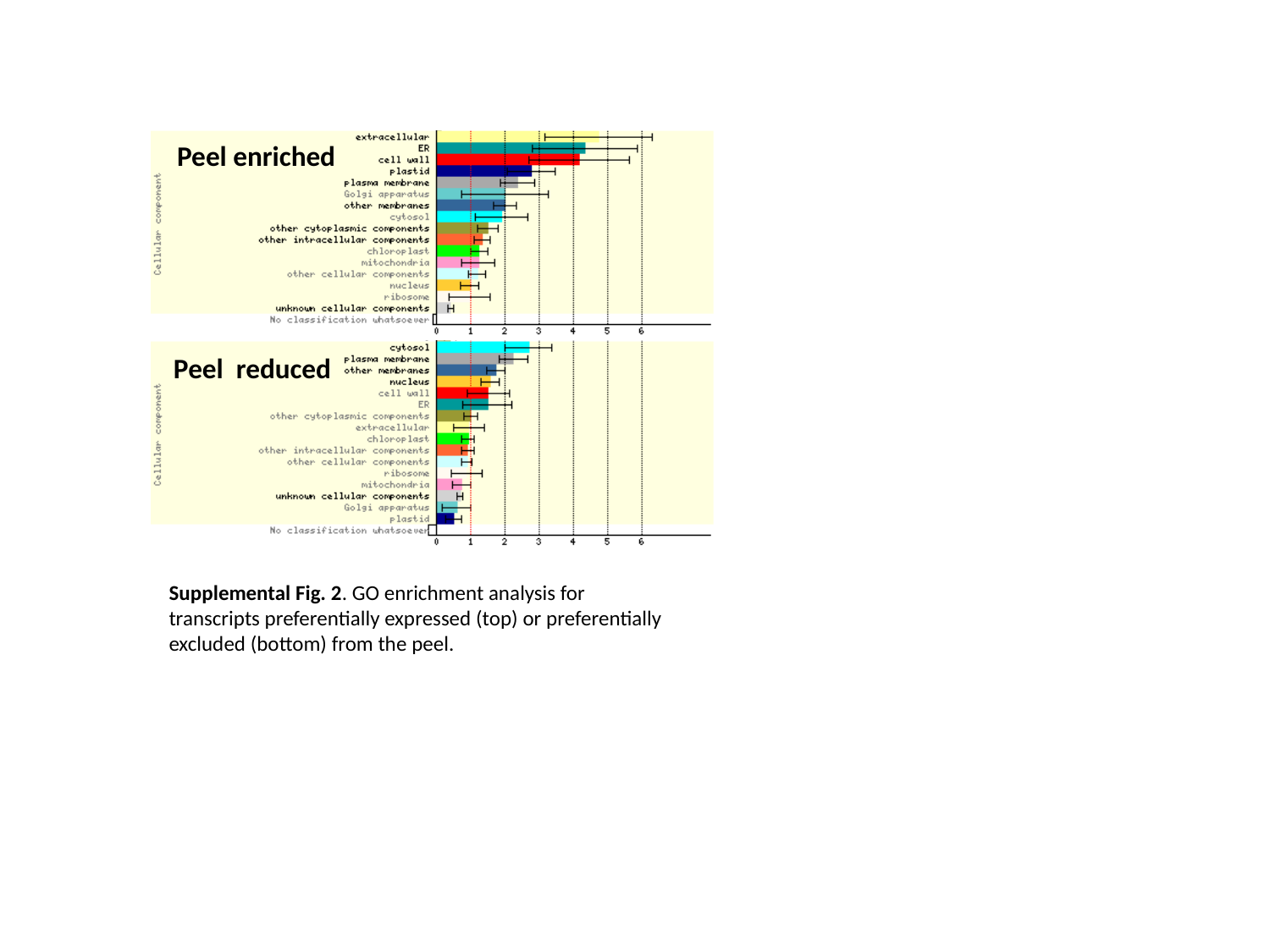

Peel enriched
Peel reduced
Supplemental Fig. 2. GO enrichment analysis for transcripts preferentially expressed (top) or preferentially excluded (bottom) from the peel.
