# Supplementary material for: Exocarp Properties and Transcriptomic Analysis of Cucumber (Cucumis sativus) Fruit Expressing Age-Related Resistance to Phytophthora capsici
Source: PLoS One. 2015 Nov 3;10(11):e0142133. doi: 10.1371/journal.pone.0142133 (PMC4631441; doi:10.1371/journal.pone.0142133)
Supplement: S1 Table — (DOCX) [file pone.0142133.s003.docx]

| Supplemental Table 1. Primers used for qRT-PCR analysis. | | | | |
| --- | --- | --- | --- | --- |
| # | Gene | Name | Forward | Reverse |
| 1 | EDS1 | EDS1 (enhanced disease susceptibility 1); lipase/ signal transducer/ triacylglycerol lipase | TGGTGAATGACAACAATGCCAC | AGGGATGCTGTTGGAATGTCA |
| 2 | ERF6 | ATERF6 (ETHYLENE RESPONSIVE ELEMENT BINDING FACTOR 6); DNA binding / transcription factor | GGGTACCGACGTTAGTTACTTGA | CGTCCCAAACCATCGACCAA |
| 3 | F3H | F3H (FLAVANONE 3-HYDROXYLASE); naringenin 3-dioxygenase | AAGGACAAAACAAACTCAATCCT | AACAGGACAGAACAAAATTGAGA |
| 4 | FLS | FLS (FLAVONOL SYNTHASE); flavonol synthase | TCAGGCGTTTAGAGATGGGC | GGATGACAAGGGCGTTAGGA |
| 5 | FMO1 | FMO1 (FLAVIN-DEPENDENT MONOOXYGENASE 1); FAD binding / NADP or NADPH binding / electron carrier/ flavin-containing monooxygenase/ monooxygenase/ oxidoreductase | ATTCACGCATCCCCAACCAC | TTTGCATGGGAAGGGACTCTC |
| 6 | GAMMA VPE | GAMMA-VPE (GAMMA VACUOLAR PROCESSING ENZYME); cysteine-type endopeptidase | CGCTAAGTGGCCAATATGCCT | AGCTGGCCTCAAGGAGTTTT |
| 7 | GDSL | GDSL-motif lipase/hydrolase family protein | AGCTGAGGGAGAAAAGATTGCT | AGGAAGCTGGAGGTTGAGTG |
| 8 | GSTU22 | ATGSTU22 (GLUTATHIONE S-TRANSFERASE TAU 22); glutathione transferase | TGGTTGAGAAGGTTGTGTGTTT | TTGCTCCTCTCCTTTTCCACC |
| 9 | KCS6 | KCS6 (3-KETOACYL-COA SYNTHASE 6); catalytic/ transferase, transferring acyl groups other than amino-acyl groups | TCTGCCAAAATTTGCTTCCTTTG | AATATATGGTGTCACAGCCCGT |
| 10 | MYB30 | MYB30 (MYB DOMAIN PROTEIN 30); DNA binding / transcription factor | GCGGCCATAGCTTCATACCT | GTGGTGGTTGTTCAGCCTCT |
| 11 | MYB96 | MYB96 (myb domain protein 96); DNA binding / transcription factor | GGGAAATGATGGGAAGGCCA | CAATTGCCAGGCCCATGTTC |
| 12 | NUDT7 | NUDT7; ADP-ribose diphosphatase/ NAD or NADH binding / hydrolase/ nucleoside-diphosphatase/ protein homodimerization | AGGCGTAGAAATGGGTTCCT | GCAGTGCCTTGAACATGGG |
| 13 | PDR12 | PDR12 (PLEIOTROPIC DRUG RESISTANCE 12); ATPase, coupled to transmembrane movement of substances | GATTGGAGCATTGGGACGGA | GGAGAGATCCAGTAGCCCCA |
| 14 | Peroxidase | peroxidase, putative | ACATAAGTTTGTAATAATGGCGGCT | AACACCATGCACAATGCTTGA |
| 15 | PUB23 | PUB23 (PLANT U-BOX 23); ubiquitin-protein ligase | TCCTGCGGTGTTGATGGAAA | CTCCTTCGTCTTGCCTCCAT |
| 16 | SANP33 | SNAP33 (SOLUBLE N-ETHYLMALEIMIDE-SENSITIVE FACTOR ADAPTOR PROTEIN 33); SNAP receptor/ protein binding | CGAGCGTTTGAAGTGAAGCAA | TGGTCAAACATTAGGGAGCGA |
| 17 | SYP121 | SYP121 (SYNTAXIN OF PLANTS 121); SNAP receptor/ protein anchor | CACCTGTCCCATTTCTCCGC | AGAAGCCAGAAGAGGGATTTGA |
| 18 | TT7 | TT7 (TRANSPARENT TESTA 7); flavonoid 3'-monooxygenase/ oxygen binding | ACCTTATAGCAGGTGGAACGG | TGCCCATTCTACTGTCACGG |
| 19 | WRKY40 | WRKY40; transcription factor | TTGACGTTTTTAGGTCGTCCAAC | AAACCCCTTGCTCCTTTGTC |
| 20 | YUC10 | YUC10; FAD binding / monooxygenase/ oxidoreductase | CCACCGTATTTGTCCTCCTCC | GTGAGGTAAGGGGTTTGGGT |
